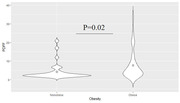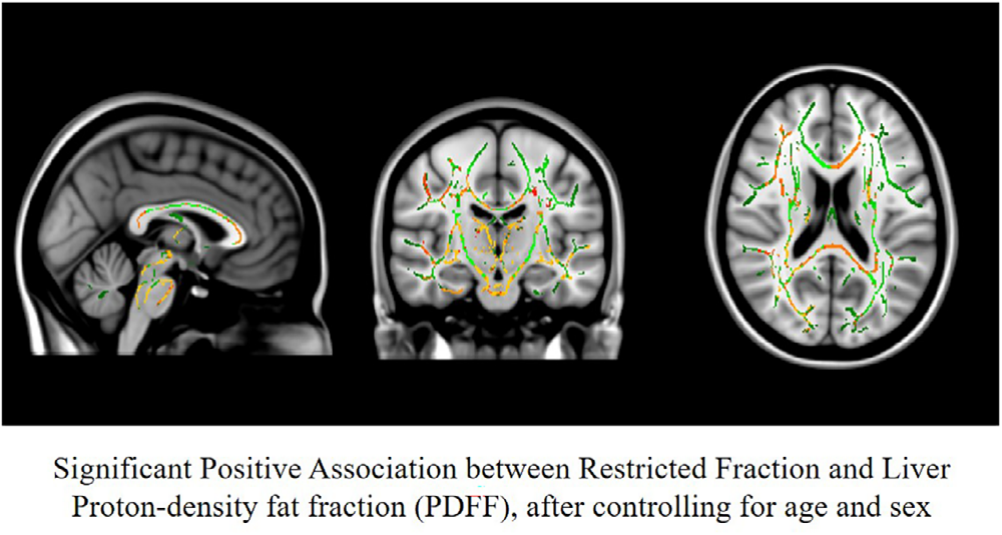# The Association between Hepatic fat and Neuroinflammation in Midlife Obesity

**DOI:** 10.1002/alz.094080

**Published:** 2025-01-09

**Authors:** Mahsa Dolatshahi, Farzaneh Rahmani, Paul K. Commean, Sara Hosseinzadeh Kasani, Mahshid Naghashzadeh, LaKisha Lloyd, Caitlyn Nguyen, Abby McBee‐Kemper, Jake Weeks, Lael Ceriani, Bettina Mittendorfer, Claude Sirlin, Sheng‐Kwei Song, Tammie L.S. Benzinger, Joseph E. Ippolito, John C. Morris, Cyrus A. Raji

**Affiliations:** ^1^ Mallinckrodt Institute of Radiology, Washington University in St. Louis, St. Louis, MO USA; ^2^ University of California, San Diego, La Jolla, CA USA; ^3^ Missouri University School of Medicine, Columbia, MO USA; ^4^ Washington University in Saint Louis, Saint Louis, MO USA; ^5^ Washington University School of Medicine in St. Louis, St. Louis, MO USA; ^6^ Washington University in St. Louis, St. Louis, MO USA; ^7^ Knight Alzheimer Disease Research Center, St. Louis, MO USA; ^8^ Washington University in St. Louis School of Medicine, St. Louis, MO USA

## Abstract

**Background:**

Obesity in midlife, body mass index (BMI) of 30 kg/m2 or higher, is recognized as a contributor to Alzheimer disease (AD) later in life. Adiposity in visceral tissues such as liver is associated with increased systemic inflammation and impaired cognition. In this study, we aimed to investigate the relationship between MRI‐derived Positron Density Fat Fraction (PDFF) and brain histology and neuroinflammation using Diffusion Basis Spectrum Imaging (DBSI) in cognitively normal midlife individuals.

**Method:**

In total, 67 cognitively normal middle‐aged participants (Age: 50.02±6.00 years, female: 65.7%, obesity: 53.7%, BMI: 31.72±6.81 kg/m2) underwent brain and abdominal 3T MRI and metabolic assessment. Homeostatic Model Assessment for Insulin Resistance (HOMAIR) was used as for measuring insulin resistance. Using a trained U‐Net convolutional neural network (CNN) model, hepatic PDFF maps were calculated from conventional T1‐weighted images. The CNN included the Adam optimizer for training. A DBSI scheme with a total of 98 diffusion samplings was acquired. After eddy current and movement correction and removing non‐brain tissue, DBSI maps including fractional anisotropy (FA, overall integrity), axial diffusivity (AD, axonal injury), radial diffusivity (RD, myelin loss), restricted fraction (RF, inflammation cellularity), hindered fraction (HF, extracellular edema), and fiber fraction (FF, axonal density) maps were calculated using in‐house software scripted in MATLAB and Statistics Toolbox Release. DBSI‐derived maps were processed using a tract‐based spatial statistics (TBSS) pipeline to allow for whole‐brain white matter voxel‐wise analyses. Using the Randomize tool from FSL, the association of PDFF and HOMAIR with DBSI‐derived white matter skeleton, with age and sex as covariates, and a threshold of 0.05 for false‐discovery rate.

**Result:**

There was a significant association between PDFF and HOMAIR (p<0.001), and obese individuals showed higher PDFF (p = 0.02) but no difference in HOMAIR (p = 0.12). We observed a significant positive association between PDFF and restricted fraction in widespread white matter tracts. There was no significant association between HOMAIR and DBSI measures.

**Conclusion:**

Our results indicate the role of hepatic fat in increased inflammation‐related cellularity in the brain in cognitively normal midlife individuals. These findings suggest that excess hepatic fat can potentially increase the risk for Alzheimer disease and cognitive impairment at least partly through promoting neuroinflammation.